# Multi-omics reveals the alleviating effect of berberine on ulcerative colitis through modulating the gut microbiome and bile acid metabolism in the gut-liver axis

**DOI:** 10.3389/fphar.2024.1494210

**Published:** 2024-10-24

**Authors:** Jingsheng Yu, Yixuan Zheng, Changmin Liu, Zhuangyuan Xie, Qingqing Liu, Shuai Yang, Qianqian Tian, Chi Song, Shilin Chen

**Affiliations:** ^1^ Institute of Chinese Materia Medica, China Academy of Chinese Medical Sciences, Beijing, China; ^2^ Institute of Herbgenomics, Chengdu University of Traditional Chinese Medicine, Chengdu, China; ^3^ Faculty of Social Sciences, The University of Hong Kong, Hong Kong SAR, China

**Keywords:** ulcerative colitis, berberine, gut microbiome, bile acid, s1pr2, intestinal barrier

## Abstract

The dysfunction of gut microbiome and bile acid metabolism might cause the incidence and relapse of ulcerative colitis (UC). Thus, natural products have been considered effective for UC through the regulation of gut microbiome and bile acid. In this study, we evaluated the regulatory effect of berberine on gut microbiome and bile acid metabolism in UC. Results showed that the relative abundances of beneficial bacteria showed a decreasing trend in the UC model, and the taurine conjugated bile acids increased from the liver tissue to the colon tissue. Berberine inhibited the colonization of harmful bacteria and promoted the primary bile acid metabolism. Moreover, we used multi-omics technology (metagenomics, metabolomics, and transcriptomics technology) to reveal that berberine restored the intestinal barrier function through bile acid/S1PR2/RhoA/ROCK pathway. The result of transmission electron microscopy directly showed that the damaged intestinal mucosal barrier was repaired through the berberine treatment. This study revealed the treatment influence on UC through multi-omics technology *in vitro* and *in vivo* models, which provides references for explaining the mechanism of berberine on UC.

## 1 Introduction

Ulcerative colitis (UC) is a common digestive system disease with relapsing and etiologically idiopathic characteristic. The clinical symptoms of UC mainly include intestinal tract bleeding, mucosal erosions, and body weight loss (Ungaro et al., 2017). Increasing number studies have demonstrated that gut microbiome and their metabolites were closely related to the development of UC. Zhang et al. (2020) transplanted gut microbiome that was obtained from healthy mice into the UC model. Results showed that this intervention decreased disease activity index (DAI) and increased the body weight and colon length. Mills et al. (2022) studied the effect of proteases of *Bacteroides vulgatus* on the disease severity of UC, and they indicated that it might prevent *B. vulgatus* colonization and improve intestinal barrier dysfunction. Thus, regulation of gut microbiome and their metabolites provides a new treatment strategy for UC. Among these metabolites, bile acid (BA) metabolism contributes to UC. BAs are steroid compounds synthesized from cholesterol in the liver and are secreted from hepatocytes into the bile canaliculi (Thomas et al., 2008). Sinha et al. (2020) compared the differences of BA between the UC and healthy control group. They indicated that gut dysbiosis caused BA deficiency, which induced pro-inflammatory in the intestine in UC patients. Meanwhile, the mechanism of BA in the UC models were widely discussed. On one hand, BAs directly affected the generation of immune cell and colon epithelial cell (Campbell et al., 2020; Yde et al., 2022). On the other, BAs were recognized as signals to regulate the relative targets and their pathways to influence UC such as Takeda G-protein receptor 5 (TGR5 or GPBAR1), Farnesoid X receptor (FXR) and sphingosine-1-phosphate receptor 2 (S1PR2, Katafuchi and Makishima, 2022; Sarkar et al., 2023; Sorrentino et al., 2020).

In recent years, more and more studies have reported that natural products exhibited potential to suppress UC. Evidence from pharmacology research showed that natural products could regulate the gut microbiome and their metabolites to affect the development of UC. Wan et al. (2024) observed that ginseng polysaccharides inhibited the expression of inflammatory cytokines in the UC model through regulating the relative abundance of *Lactobacillus* and tryptophan metabolism. Li et al. (2022) studied the mechanism of Huangqin decoction treatment on UC in the dextran sulfate sodium (DSS) induced mice model. Results showed that this decoction mainly restored the gut microbiome balance and upregulated amino acid metabolism to activate the mTOR signaling pathway. Lee et al. (2018) reported that camellia oil showed the inhibitory effect on UC. The relative abundance of Bifidobacterium in the camellia oil group was lower than that in the DSS model group. Meanwhile, camellia oil increased antioxidant and antioxidant enzyme activities and reduced inflammatory damage. Based on the previous studies, natural products have the potential to be explored into candidate therapy drug for UC.

In this study, we analyzed the therapy effect of berberine, an isoquinoline alkaloid originated from *Coptis chinensis* Franch., *Phellodendron chinense* C. K. Schneid., and *Mahonia fortunei* (Lindl.) Fedde on the DSS-induced UC models. Multi-omics technologies (transcriptomics, metagenomics, and metabolomics) were combined to reveal the interaction between gene expression, gut microbiome, and BAs during the intervention of berberine in the UC mice models. This study aims to systematically reveal the therapy role of berberine in UC, which provides references for the treatment of UC using natural products.

## 2 Materials and methods

### 2.1 Reagents and chemicals

Dextran sulfate sodium (36–50 kDa, colitis grade) was purchased from MP Biomedicals (Shanghai, China). Berberine (>98% purity, 141,433-60-5) was obtained from TopScience Co., Ltd. (Shanghai, China). The fecal occult blood test kit was obtained from MLBio Co. (Shanghai, China). The dimethylsulfoxide (CAS 67-68-5, Beijing, China) was obtained from Solarbio Co., Ltd. The hematoxylin and eosin (H&E) staining kit (Cat DH0020, Leagene, Beijing, China) and xylene (No. 2022051901, Chron chemicals, Chengdu, China) were used for H&E staining. The JTE 013 (antagonist for S1PR2, CAS 383150-41-2) and CYM-5520 (agonist for S1PR2, CAS 1449747-00-5) were purchased from Selleck company (Shanghai, China). The S1PR2 and GAPDH specific primary antibodies (S1PR2, Cohesion Biosciences, Shanghai, China; GAPDH, Abcam) were purchased from Cohesion Biosciences and Abcam, respectively. The HRP* goat anti rabbit IgG antibodies secondary antibody was purchased from Immunoway Biotechnology Company (Suzhou, China). The xylene and rehydration with gradient ethanol (No. 2022070501) were purchased from the Chron chemicals company (Chengdu, China). The ECL Western blotting Substrate was purchased from the Solarbio company (China).

### 2.2 Animals and DSS-induced UC experiment

Male C57BL/6 mice (eight-week-old, 25 ± 1 g) were obtained from Chengdu Dossy Experimental Animals Co., Ltd. (Chengdu, China). All animals were maintained under specific pathogen-free (SPF) conditions with 12 h of light/12 h of dark cycle. The experiments were approved by the Bioethics Committee of the Chengdu Dossy Experimental Animals Co., Ltd. (IACUC-SWLAB-2024020101). The DSS was dissolved in sterile water to prepare 2.5% DSS solution and the solution was further filtered using 0.22 μm millipore filter membrane. Six milligrams of berberine were dissolved in 150 μL of dimethyl sulfoxide. The 150 μL of solution was transported in a 5 mL tube. Then, we added 150 μL of Tween-80 (Cas. 25322-68-3, Solarbio, Beijing, China) into the solution. A total of 1800 μL sterile water was used to dilute the solution. The solution was further filtered using 0.22 μm millipore filter membrane for gavage. After 1 week of adaptive feeding, all mice were randomly divided into five groups including control (normal saline once a day, n = 5, BerC), model (2.5% DSS + normal saline once a day, n = 5, BerM), low-level berberine treated (2.5% DSS+10 mg/kg berberine once a day, n = 5, BerL), high-level berberine treated (2.5% DSS+50 mg/kg once a day, n = 5, BerH), and 5-Aminosalicylic Acid groups (5-ASA, Cas. 89-57-6, TopScience Co., Ltd., Shanghai, China, 2.5% DSS+100 mg/kg once a day, n = 5, BerP).

### 2.3 Morphological and histopathological examination

The mouse fresh colon tissues were fixed using 4% paraformaldehyde for more than 24 h and embedded in paraffin with a series of ethanol dilutions and sectioned into 3 μm slides. The slides were stained using a H&E staining kit after deparaffinization with xylene and rehydration with gradient ethanol. The result was observed using the fluorescence microscope (PanoScanner 20, PanoVue, Beijing, China).

### 2.4 Construction of S1PR2/HEK293T stable cell line

The HEK293T cells were cultured in DMEM medium (LOT 11995500BT, ThermoFisher, Shanghai, China) with 10% FBS (LOT 2401119, VivaCell BIOSCIENCES, Shanghai, China) under proper conditions (37°C; 5% CO_2_). The construction protocol was performed based on Yang et al. (2024). The pCDH-CMV-S1PR2 (human)-EF1-CopGFP-T2A-Puro plasmid was used to construct overexpression cell line. This cell line was applied to construct a S1PR2 overexpression stable cell line through the Lipofectamine 3,000 Kit (Invitrogen, Unites states). The cells were transferred into 6-well plates with 3.5 × 10^5^ per plate and incubated for 48–72 h in DMEM with 10% FBS and Puromycin solution (2 μg/mL, No. ST551, Beyotime Inc.). Then, the purified cells were incubated with fresh medium containing 1 μM of JTE 013 (antagonist for S1PR2, CAS 383150-41-2, Selleck, America) or 1 μM of CYM-5520 (agonist for S1PR2, CAS 1449747-00-5, Selleck, America) for 24 h. The S1PR2/HEK293T stable cell lines and their medium supernatant were obtained to perform further study.

### 2.5 Quantitative real-time -PCR

The RNA products of S1PR2/HEK293T stable cell line were extracted using the RNA isolater Total RNA Extraction Reagent (Vazyme, Nanjing, China). We performed the reverse transcription protocol using the RT Easy™ II kit instruction (RT-01022, Foregene Co., Ltd., Chengdu, China). We further used Genious 2X SYBR Green Fast qPCR Mix (RK21205, ABclonal, Wuhan, China) to perform qRT-PCR. The mRNA expression of S1PR2 and its downstream RhoA, ROCK, ZO-1, E-cadherin, and Occludin genes were evaluated by the 2^-△△Ct^ method and normalized with GAPDH. The primer sequences were designed in Sangon Biotech (Sangon, Shanghai, China, Table 1).

**TABLE 1:** Designed primer information in this study.

Primers	DNA sequence
S1PR2F	5′-CCA​CCA​CCT​CCT​GCC​ACT​CC-3′
S1PR2R	5′-CAC​CGT​GTT​GCC​CTC​CAG​AAA​C-3′
ZO-1F	5′-GCG​GAT​GGT​GCT​ACA​AGT​GAT​G-3′
ZO-1R	5′-GCC​TTC​TGT​GTC​TGT​GTC​TTC​ATA​G-3′
E-CadherinF	5′-TCT​GCT​GCT​CTT​GCT​GTT​TCT​TC-3′
E-CadherinR	5′-TCT​CCG​CCT​CCT​TCT​TCA​TCA​TAG-3′
OccludinF	5′-ATT​AAC​TTC​GCC​TGT​GGA​TGA​CTT​C-3′
OccludinR	5′-TCT​TGC​TCT​GTT​CTC​TTT​GAC​CTT​C-3′
RhoAF	5′-AGG​TGG​ATG​GAA​AGC​AGG​TAG​AG-3′
RhoAR	5′-AGT​ATA​ACA​TCG​GTA​TCT​GGG​TAG​GAG-3′
GAPDHF	5′-GGA​GCG​AGA​TCC​CTC​CAA​AAT-3′
GAPDHR	5′-GGC​TGT​TGT​CAT​ACT​TCT​CAT​GG-3′
ROCKF	5′-TGC​CGC​CGT​TGC​CAT​ATT​AAG-3′
ROCKR	5′-CCA​CTT​CTG​CTG​CTC​TTC​TGT​AG-3′

### 2.6 Western blot assay

We extracted the protein products from the cell and tissue samples using RIPA buffer with 1% PMSF as the kit instruction (R0020, Solarbio, China). The quantity analysis of protein concentration was performed based on the instruction of the BCA Protein assay kit (P0010, Beyotime, Shanghai, China). We separated the protein products with a sodium dodecyl sulfate–polyacrylamide gel electrophoresis gel. Then, we used the S1PR2 and GAPDH specific primary antibodies to incubate the membrane at 4°C overnight. The HRP* goat anti rabbit IgG antibodies was applied as secondary antibody to incubate membrane and the ECL Western blotting Substrate was used to detect the protein blots.

### 2.7 Metagenomic analysis of gut microbiome

We collected 100 mg of fresh fecal samples at various days and frozen at −80°C immediately. Then, we extracted the DNA products as the TIANamp Stool DNA KIT instruction (DP328, TIANGEN Bio Tech Co., Ltd., Beijing, China). The DNA product was sequenced on the Illumina NovaSeq 6,000 platform. All data was uploaded to the National Center for Biotechnology Information Sequence Read Archive database with accession numbers. The low-quality data was filtered using fastp and Samtools software. We used Megahit software (v. 1.2.9, https://github.com/voutcn/megahit) to assemble the contigs. The Kraken2 software was applied to taxonomy annotation. We assessed the bacterial community diversity using the alpha and beta diversity through QIIME2 of R package. The Venn diagram and heatmap were performed using R package. The Principal Component Analysis and Principal coordinates analysis were conducted using R package.

### 2.8 Transcriptomics profiling of gene expression in colon tissues

Approximately 150 mg fresh colon tissue samples were griding in liquid nitrogen and transferred into centrifuged tubes with 500 µL RB Buffer. The samples were transferred into gDNA Filter and centrifuged at room temperature with 14,000 × g for 5 minutes. The filtered solution was mixed with 250 µL ethyl alcohol and vortexed for 20 s. The filtered solution was transferred into HiBind ^®^ RNA Mini Filter and centrifuged at room temperature with 12,000 × g for 1 minute. The RNA products were washed with RWF wash Buffer and RNA wash Buffer II. The quality of RNA products was evaluated using NanoDrop One spectrophotometer (NanoDrop Technologies, Wilmington, DE) and Qubit 3.0 Fluorometer (Life Technologies, Carlsbad, CA, United States). The sequencing was performed on the DNBseq platform. The sequencing quality of raw data was assessed using Fast QC (v. 0.11.9, https://www.bioinformatics.babraham.ac.uk/projects/fastqc). The clean reads, Q20, Q30, and GC content were calculated. The DESeq2 (v.1.26.0) was applied to analyze differentially expressed genes (DEGs) with *p*-value below 0.05 and |log_2_Fold Change|>1. The Gene Ontology (GO, v. 2022-03-09, http://geneontology.org/) enrichment and Kyoto Encyclopedia of Genes and Genomes (KEGG, 2021-05-03, https://www.kegg.jp/kegg/) enrichment was performed based on the genes identified in this study with clusterProfiler software (v. 3.14.3). The sample correlation was calculated with pearson correlation coefficient >0.9. The volcano plot was performed using Prism 9 software.

### 2.9 Metabolomic analysis of bile acid in the fecal and liver tissue samples

#### 2.9.1 Sample pretreatment

Approximately 20 mg of fecal or colon samples were transferred with one zirconium bead. Ten microliters of the internal standard (1 μg/mL) and 500 μL methanol were added and homogenated. The solution was shaken for 10 minutes (2,500 r/min) and stored at −20°C for 10 minutes. The samples were centrifuged for 10 minutes at 4°C (12000 r/min) and filtered.

#### 2.9.2 LC-MS/MS detection

The analysis was performed on the ultra-performance liquid chromatography (ExionLC™ AD) coupled with Tandem Mass Spectrometry (MS/MS, QTRAP^®^ 6,500+). The chromatographic separation was conducted on a Waters ACQUITY UPLC HSS T3-C_18_ column (2.1 mm × 100 mm, 1.8 μm, Waters, Milford, MA, United States). Mobile phases consisted of 0.01% acetic acid and 5 mmol/L ammonium acetate in water (phase A, v/v) and 0.01% acetic acid in acetonitrile (phase B, v/v). The flow rate was 0.35 mL/min. The gradient program was as follows: 0 min A/B (95:5), 0.5 min A/B (60:40), 4.5 min A/B (50:50), 7.5 min A/B (25:75), 10 min A/B (5:95), 12 min A/B (95:5). The electrospray ionization (ESI) temperature was 550°C.

### 2.10 Transmission electron microscopy for colon tissue

The colon tissues were fixed in 2.5% glutaraldehyde (pH 7.4, No. 18427, Ted Pella Inc., Shanghai, China) for at least 72 h. The detailed protocol was performed as Wang et al. (2024) described. The colon tissue slices were observed with a transmission electron microscope (JEM1400, JEOL Ltd., China).

## 3 Results

### 3.1 Berberine suppressed UC in dextran sulfate sodium induced mice

**FIGURE 1:**
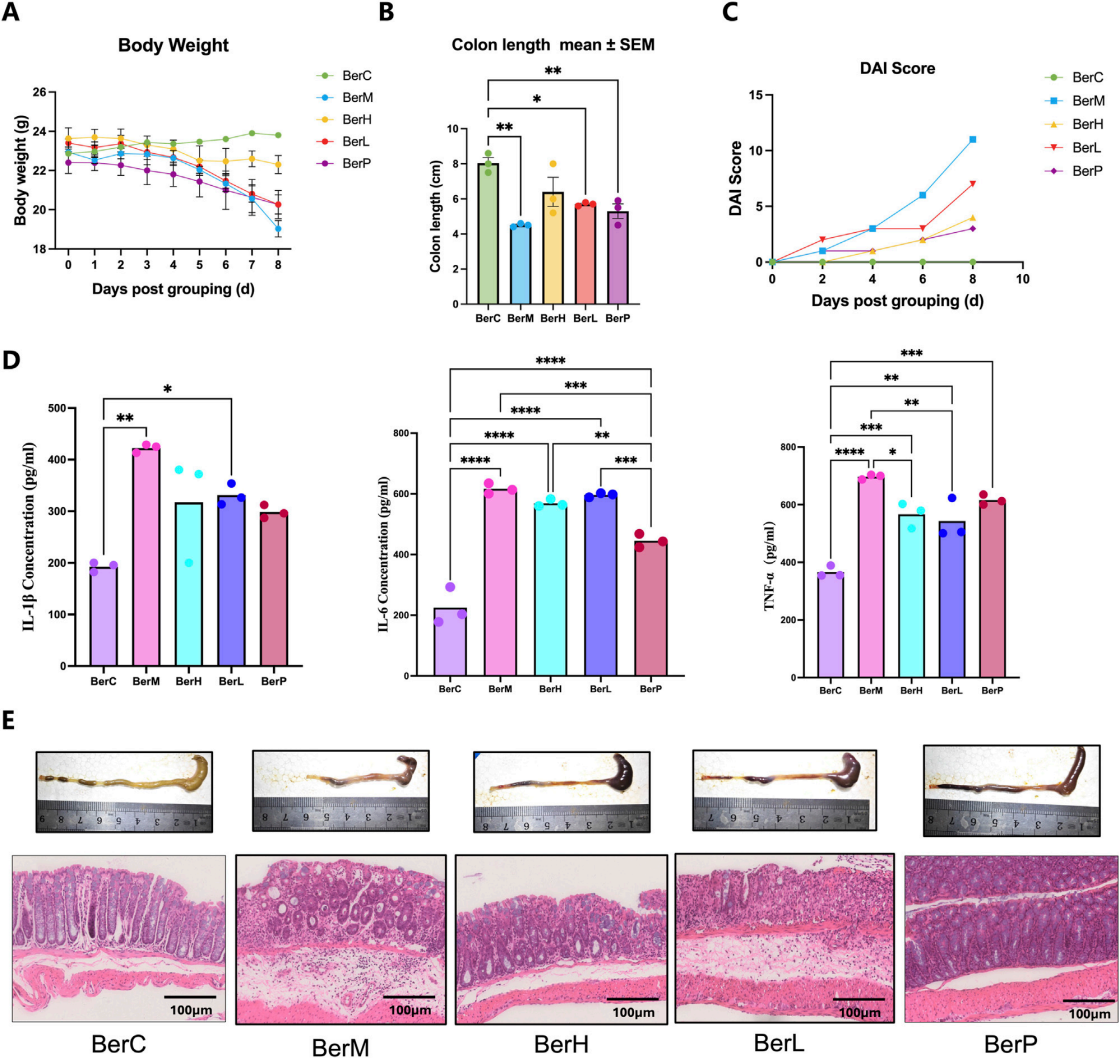
Berberine suppressed UC in DSS induced model. **(A)** Body weight monitor in the BerM, BerL, BerH, BerP, and BerC groups; **(B)** Colon length in the BerM, BerL, BerH, BerP, and BerC groups; **(C)** DAI score changes in the BerM, BerL, BerH, BerP, and BerC groups; **(D)** Determination of three pro-inflammatory cytokine level (TNF-α, IL-1β, and IL-6) in serum samples in the BerM, BerL, BerH, BerP, and BerC groups; **(E)** Representative photomicrographs of H&E staining and quantification of colon tissue in the BerM, BerL, BerH, BerP and BerC groups. *represents *p* < 0.05, ** represents *p* < 0.01, *** represents *p* < 0.001, **** represents *p* < 0.0001.

In this study, we used various concentrations of berberine (10 and 50 mg/kg) to treat DSS-induced mice. Results showed that berberine alleviated certain pathological symptoms of UC mice model. The body weight in the BerM group was lower than that in the other groups and berberine treatment suppressed the body weight loss of model animals (Figure 1A). The result showed that the colon length in the BerM group was significantly shorter than that in the other groups (*p* = 0.0023, Figure 1B). DAI index in the BerM group was highest, followed by the BerL, BerH, BerP and BerC group (Figure 1C). We also determined the contents of three main cytokines (TNF-α, IL-1β, and IL-6) in serum in various groups. We observed that berberine remarkably inhibited the production of TNF-α, IL-1β, and IL-6 (Figure 1D). It also alleviated the intestinal tract bleeding and mucosal erosions in DSS induced models based on the histopathological evaluation (Figure 1E).

### 3.2 Metabolomics profiling revealed the bile acid composition in the liver tissue

**FIGURE 2:**
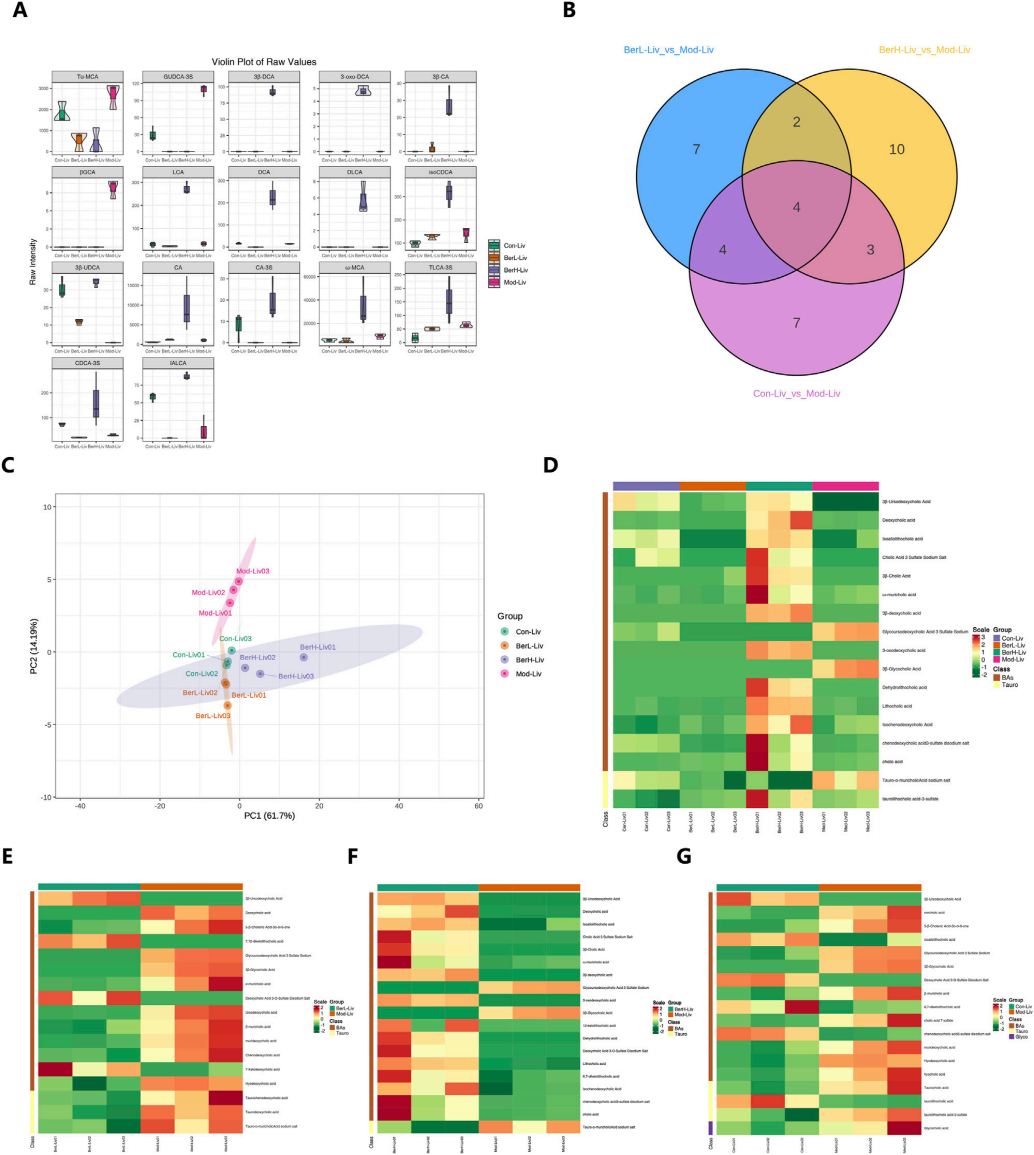
Metabolome profiling of bile acids in liver tissue samples. **(A)** Comparison of bile acids in liver tissues in the BerM, BerL, BerH, and BerC groups based on the full violin diagrams; **(B)** Venn diagram reveal the unique and shared bile acid numbers in the BerM, BerL, BerH, and BerC groups; **(C)** Principal Component Analysis of bile acid composition in the BerM, BerL, BerH, and BerC groups; **(D)** Heatmap analysis of bile acid differences in the BerM, BerL, BerH, and BerC groups; **(E)** Differences of bile acid between the BerM and BerL group; **(F)** Differences of bile acid between the BerM and BerH group; **(G)** Differences of bile acid between the BerM and BerC group.

In this study, we used bile acid-targeted metabolomics technology to detect the bile acid level in the liver. Results showed that the level of 17 bile acid and associated products in various groups were significantly different including 3β-ursodeoxycholic acid (3β-UDCA), deoxycholic acid (DCA), isoallolithocholic acid (IALCA), cholic acid 3 sulfate sodium salt (CA-3S), 3β-cholic acid (3β-CA), ω-muricholic acid (ω-MCA), 3β-deoxycholic acid (3β-DCA), glycoursodeoxycholic acid 3 sulfate sodium (GUDCA-3S), 3-oxodeoxycholic acid (3-oxo-DCA), β-glycocholic Acid (βGCA), dehydrolithocholic acid (DLCA), lithocholic acid (LCA), isochenodeoxycholic acid (isoCDCA), chenodeoxycholic acid 3-sulfate disodium salt (CDCA-3S), cholic acid (CA), tauro-α-muricholic acid sodium salt (Tα-MCA) and taurolithocholic acid-3-sulfate (TLCA-3S, Figure 2A). Venn diagram showed that the numbers of different bile acids between various groups were different (Figure 2B). There were six shared bile acids between Mod_Liv_ VS_BerL_Liv and Mod_Liv _VS_BerH_Liv; there were eight shared bile acids between Mod_Liv_VS_BerL_Liv and Mod_Liv_VS_Con_Liv; there were seven shared bile acids between Mod_Liv_VS_BerH_Liv and Mod_Liv_VS_Con_Liv. The differences of bile acid between various groups were observed based on the PCA result (Figure 2C). We further compared the differences of bile acid between various groups (Figures 2D, E). We evaluated the effect of low-level berberine on the bile acid metabolism changes. Results showed that low-level berberine inhibited the accumulation of taurine conjugated bile acids including taurochenodeoxycholic acid (TCDCA), taurodeoxycholic acid (TDCA) and tauro-α-muricholic acid sodium salt (Tα-MCA) in the BerL group. Meanwhile, low-level berberine inhibited the accumulation of certain bile acids such as DCA, α-muricholic acid (α-MCA), β-muricholic acid (β-MCA), murideoxycholic acid (MDCA), while it promoted the accumulation of βGCA, 5-β-cholanic acid-3α-ol-6-one (6-ketoLCA), deoxycholic acid 3-O-sulfate disodium salt (DCA-3-O-S), and 7-Ketodeoxycholic acid (7-KDCA). Additionally, high-level berberine also restored the bile acid metabolism in the liver. The levels of certain bile acids including 3β-DCA, DLCA, and DCA were higher in the BerH group than in the BerM group. The tauro-α-muricholic acid sodium salt level in the BerM group was higher than that in the BerH group. Compared with the BerC and BerM group, berberine affected the metabolism of bile acid in the liver (Figures 2D, F, G).

### 3.3 Gut bacterial community composition and diversity in various groups

**FIGURE 3:**
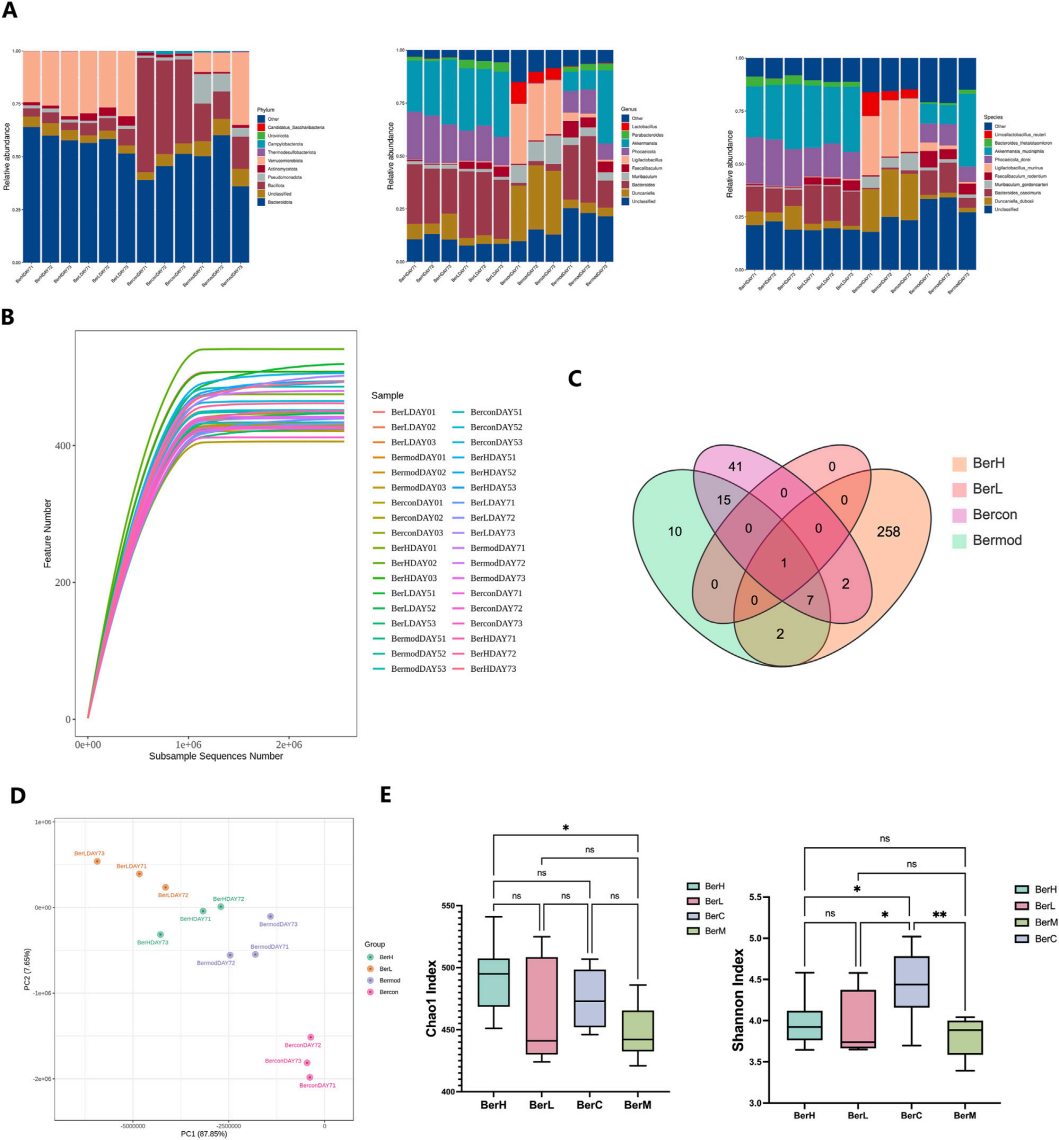
Metagenome sequencing of gut bacterial community in fecal samples. **(A)** The relative abundances of ten dominant bacteria at the phylum, genus, and species level in the BerM, BerL, BerH, and BerC groups at DAY7; **(B)** Rarefaction curve reflects sequencing depth in the BerM, BerL, BerH, and BerC groups; **(C)** Venn diagram reveal the unique and shared taxa numbers in the BerM, BerL, BerH, and BerC groups; **(D)** Principal Component Analysis of bacterial community difference in the BerM, BerL, BerH, and BerC groups; **(E)** Alpha diversity indices of Chao1 and Shannon in the BerM, BerL, BerH, and BerC groups.

We revealed the gut bacterial community composition through metagenomics technology. At the phylum level, Bacteroidota, Bacillota, and Pseudomonadota were dominant among all phyla. Further taxonomy at the class level showed that the relative abundances of Bacteroidia, Clostridia, and Erysipelotrichia were higher than other classes. Bacteroidales featured highest relative abundance among all orders. At the family level, Muribaculaceae, Bacteroidaceae, and Lachnospiraceae were dominant. *Duncaniella*, *Bacteroides*, and *Muribaculum* featured high relative abundances in this study at the genus level. *Duncaniella dubosii*, *Bacteroides caecimuris*, and *Muribaculum gordoncarteri* had the highest relative abundances at the species level (Figure 3A). Rarefaction curve result showed that the sequencing depth was sufficient to reflect the gut bacterial community (Figure 3B). The Venn diagram demonstrated that certain gut microbiome were shared in various groups despite most of them showing various change trends (Figure 3C). PCA analysis also showed that the gut microbiome exhibited differences between various groups (Figure 3D). Additionally, we assessed the bacterial community diversity and richness between the four groups based on the Chao1 and Shannon indices. The results showed these two indices were both lower in the BerM group than those in the other groups, while the Chao1 index was highest in the BerC group. The Shannon index was detected highest in the BerL group (Figure 3E).

### 3.4 Monitoring the change of gut microbiome during the berberine treatment

**FIGURE 4:**
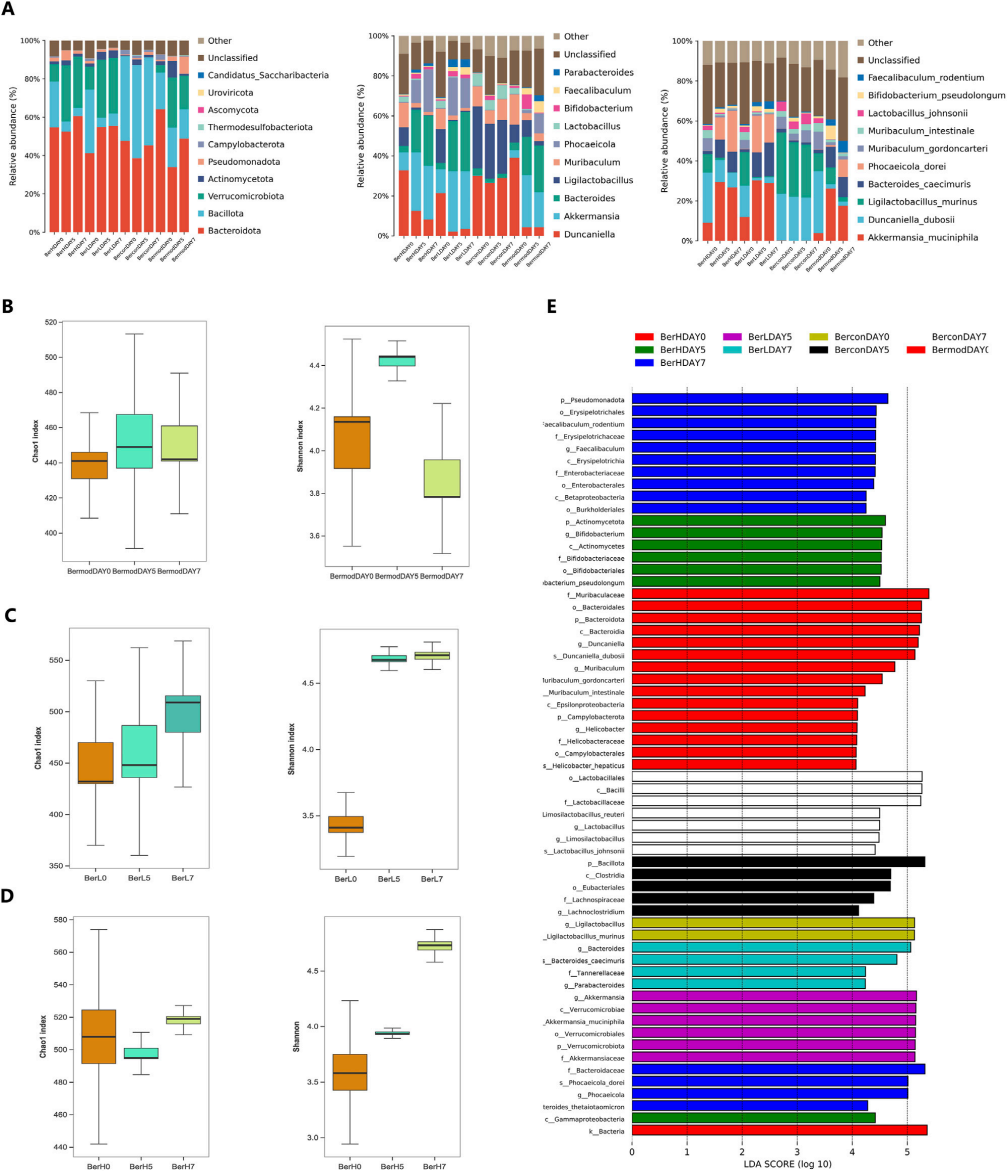
Comparison of gut bacterial community differences in fecal samples in different groups. **(A)** The relative abundances of ten dominant bacteria at the phylum, genus, and species level in the BerM, BerL, BerH, and BerC groups; **(B)** Alpha diversity indices of Chao1 and Shannon at different stages in the BerM group; **(C)** Alpha diversity indices of Chao1 and Shannon at different stages in the BerL group; **(D)** Alpha diversity indices of Chao1 and Shannon at different stages in the BerH group; **(E)** Linear discriminant analysis effect size analysis of differences of bacterial community in the BerM, BerL, BerH, and BerC groups.

To monitor the dynamic changes of gut microbiome during the berberine treatment, we analyzed the gut bacterial community in the BerL, BerH, BerC, and BerM groups. At the phylum level, Bacteroidia was dominant in all groups. The relative abundance of Bacillota was lower in the BerL and BerH group than that in the other two groups. At the class level, the relative abundance of Bacteroidia showed an increasing trend in the berberine treatment group (BerL and BerH), while it showed a decreasing trend in the BerM group. The relative abundance of Bacilli in the BerL, BerM and BerH group showed a decreasing trend, and it remained a stable level in the BerC group. At the order level, Bacteroidales was the most abundant among all orders. The relative abundance of Verrucomicrobiales showed an increasing trend in the BerL, BerM and BerH group, while it was significantly lower in the BerC group compared with other groups. However, Lactobacillales had high relative abundance in the BerC group. At the family level, the relative abundances of Muribaculaceae and Lactobacillaceae at DAY0 were significantly higher than at DAY5 and DAY7 in the BerL, BerM and BerH group. Akkermansiaceae also featured high relative abundance in these three groups. At the genus level, the relative abundances of *Akkermansia*, *Bacteroides*, and *Phocaeicola* increased from DAY0 to DAY7in the berberine treatment group (BerL and BerH), while *Ligilactobacillus* and *Muribaculum* showed decreasing trend in these two groups. At the species level, *Akkermansia muciniphila* was detected in the BerL, BerM and BerH group. The distribution of *Duncaniella dubosii*, *Ligilactobacillus murinus*, and *B. caecimuris* were detected in all groups. *Phocaeicola dorei* featured higher relative abundance in the berberine treatment group at late stage (Figure 4A). Additionally, we calculated the change of alpha diversity indices (Chao1 and Shannon) in the BerL, BerM and BerH group. Results showed that both alpha diversity indices showed an increasing trend in the BerL and BerH group, while these two indices showed decreasing trend in the BerM group (Figures 4B–D). We further used lefse software to analyze the different taxonomy in various groups and observed that the relative abundances of *Bacteroides* and *Faecalibaculum* were significantly higher in the BerH group, and *Akkermansia* featured higher relative abundance in the BerL group. *Duncaniella* and *Muribaculum* featured higher relative abundance in the BerM group (Figure 4E).

### 3.5 Metabolomics profiling revealed the bile acid composition and changes in the fecal samples during the berberine treatment

**FIGURE 5:**
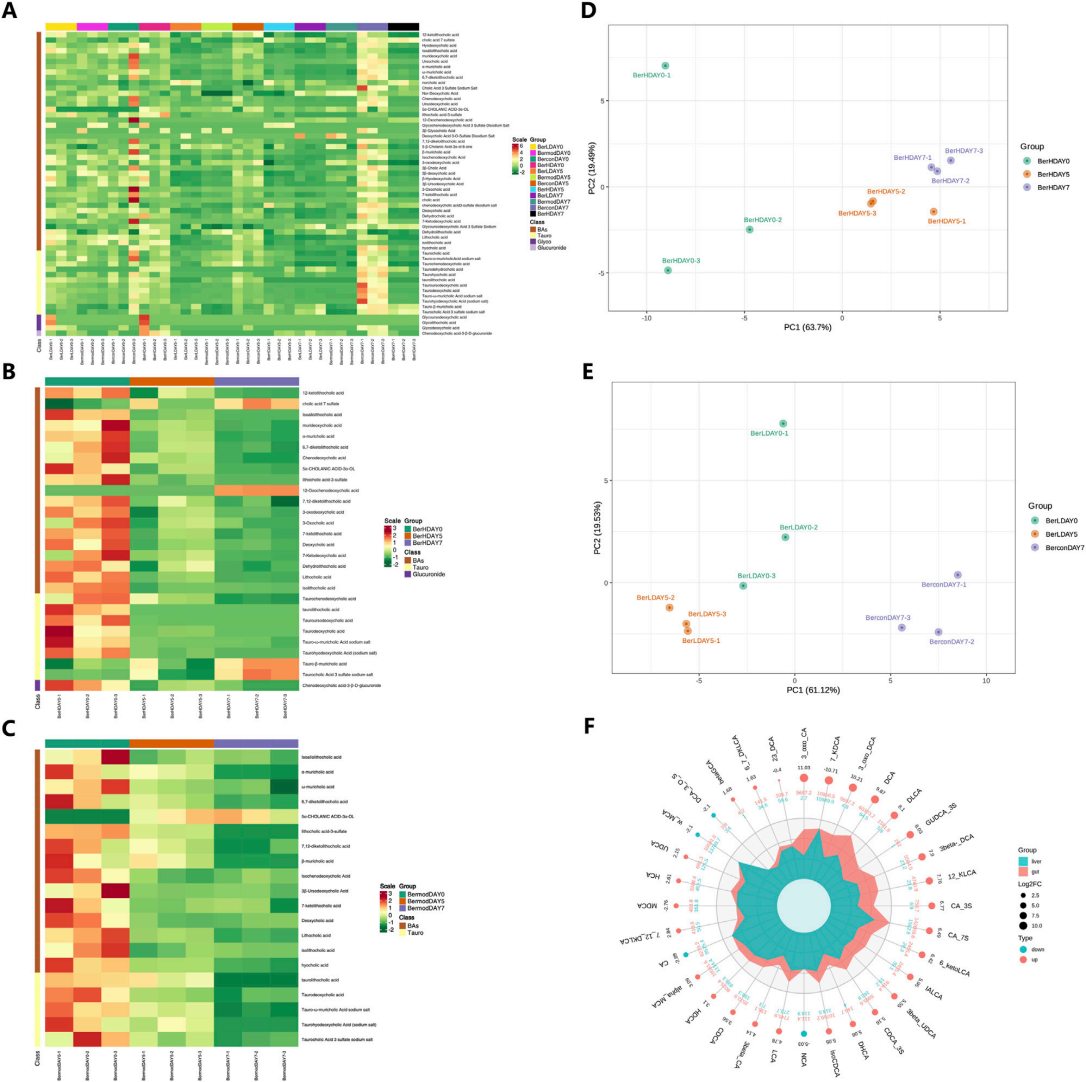
Metabolome profiling of bile acids in fecal samples. **(A)** Comparison of bile acids in different groups based on heatmap; **(B)** Bile acid differences at different stages in the BerH group; **(C)** Bile acid differences at different stages in the BerM group; **(D)** Principal Component Analysis of bile acid composition in the BerH group; **(E)** Principal Component Analysis of bile acid composition in the BerL group; **(F)** Comparison of bile acid metabolism between the liver and colon tissue.

We determined the changes of bile acids in the fecal samples at DAY0, DAY5 and DAY7. Among the 65 detected bile acids and their associated products, a total of 58 bile acids showed significant differences between various groups (Figure 5A). We further evaluated the effect of berberine on the bile acid metabolism. In the BerH group, berberine decreased most bile acid metabolisms including 12-ketolithocholic acid (12-KLCA), IALCA, MDCA, α-MCA, 6,7-diketolithocholic acid (6,7-DKLCA), chenodeoxycholic acid (CDCA), 5α-CHOLANIC ACID-3α-OL (alloLCA), TLCA-3S, 7,12-diketolithocholic acid (7,12-DKLCA), 3-oxo-DCA, 3-oxocholic acid (3-oxo-CA), 7-ketolithocholic acid (7-KLCA), DCA, 7-KDCA, DLCA, LCA, isolithocholic acid (ILCA), chenodeoxycholic acid-3-β-D-glucuronide (CDCA-3Gln), TCDCA, TDCA, tauro-ω-muricholic acid sodium salt (Tω-MCA), and taurohyodeoxycholic acid (THDCA), while caused the accumulation of cholic acid 7 sulfate (CA-7S), 12-oxochenodeoxycholic acid (12-oxo-CDCA), tauro-β-muricholic acid (Tβ-MCA) and taurocholic acid 3 sulfate sodium salt (TCA-3S, Figure 5B). In the BerM group, the level of alloLCA showed an increasing trend, while other bile acids showed decreasing trends (Figure 5C). The PCA result also demonstrated the bile acid metabolism was affected in the DSS-induced model and berberine might affect the gut microbiome and bile acid metabolism to suppress UC development (Figures 5D, E).

### 3.6 Comparison of bile acid metabolism between the liver and colon tissue

We further compared the differences of bile acids between the liver and colon tissue samples using R software (version 4.2.0, Figure 5F). The changes of 32 bile acids and their associated products were observed between the liver and colon tissue samples. A total of 28 compounds showed higher level in the colon level than in the liver tissue including 2,3-DCA, 3-oxo-CA, 3β-UDCA, 6-ketoLCA, IALCA, 7,12-DKLCA, CA-3S, DHCA, 3β-CA, DCA, 3β-DCA, GUDCA-3S, 3-oxo-DCA, β-GCA, 12-KLCA, DLCA, α-MCA, LCA, UCA, UDCA, β-MCA, 6,7-DKLCA, CA-7S, isoCDCA, CDCA-3S, MDCA, CDCA, 7-KDCA, HDCA, and HCA, while the colon tissue featured lower level of NCA, ω-MCA, DCA-3-O-S, and CA.

### 3.7 Transcriptomics sequencing of gene expression in the colon tissue

**FIGURE 6:**
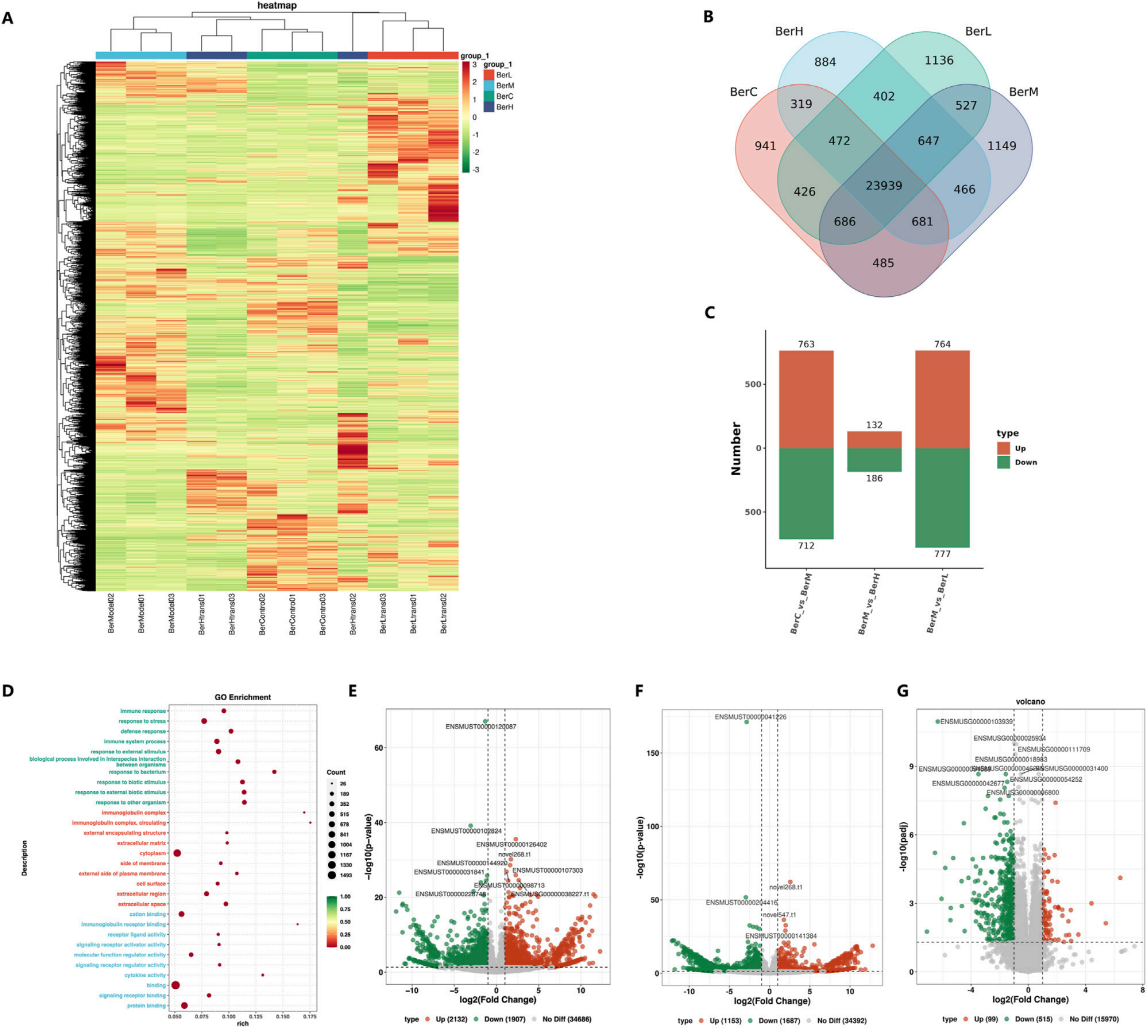
Transcriptome sequencing of gene expression in colon tissues. **(A)** Comparison of expression level in the BerM, BerL, BerH, and BerC groups based on heatmap; **(B)** Venn diagram reveal the unique and shared gene numbers in the BerM, BerL, BerH, and BerC groups; **(C)** The numbers of upregulated and downregulated genes in the BerM, BerL, BerH, and BerC group; **(D)** Gene function prediction of the transcriptome data based on the Gene Ontology database; **(E)** Comparison of differentially expressed genes in the BerL and BerM group; **(F)** Comparison of differentially expressed genes in the BerH and BerM group; **(G)** Comparison of differentially expressed genes in the BerC and BerM group.

A total of 12 samples were used for transcriptomics sequencing and the clean read numbers ranged from 43,313,658 to 43,382,416. The GC contents ranged from 45.5% to 47.5% among all samples. The heatmap results showed that the gene expression levels in the same group were similar, while significant differences were observed between different groups (Figure 6A). The DEG numbers were as follows: BerC group (87287 genes), BerH group (85220 genes), BerL group (88206 genes), and BerM group (89020 genes) based on the Venn result (Figure 6B). Compared with the BerM group, a total of 763 and 712 genes were upregulated expressed and downregulated expressed in the BerC group; a total of 186 and 132 genes were upregulated expressed and downregulated expressed in the BerH group; a total of 777 and 764 genes were upregulated expressed and downregulated expressed in the BerL group (Figure 6C). KEGG function enrichment analysis showed that the inflammatory associated pathway such as immune response, response to stress, defense response, and immune system process were affected during the berberine treatment process (Figure 6D). According to the volcano diagrams, the expression levels of S1PR2 and its downstream RhoA/ROCK were lower in the berberine groups (BerH, BerC, and BerL group) than in the BerM group (Figures 6E–G). Based on the transcriptomics sequencing results, berberine treatment remarkably influenced the inflammatory and intestinal barrier associated pathway.

### 3.8 Berberine restored the intestinal barrier function through regulation of S1PR2/cAMP/RhoA/ROCK pathway

**FIGURE 7:**
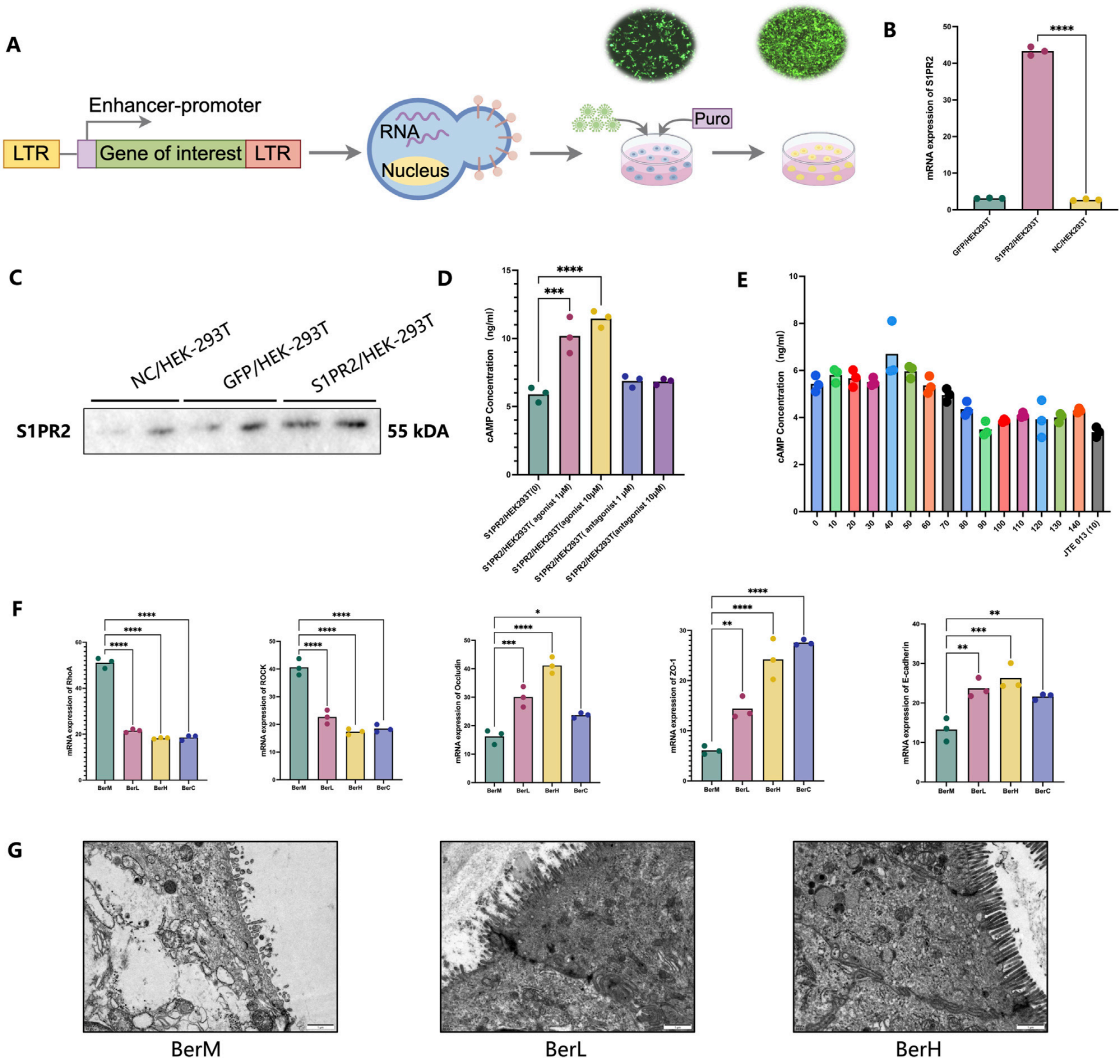
The restoring effect of berberine on the intestinal barrier function *in vitro* and *in vivo* models. **(A)** Illustrating the construction of the S1PR2/HEK293T overexpression stable cell line; **(B)** mRNA expression level of S1PR2 in the S1PR2/HEK293T overexpression stable cell line, GFP/HEK293T stable cell line, and HEK293T cell line based on qPCR results; **(C)** protein expression level of S1PR2 in the S1PR2/HEK293T overexpression stable cell line, GFP/HEK293T stable cell line, and HEK293T cell line based on WB results; **(D)** cAMP concentration determination by ELISA methods in the treatment of S1PR2/HEK293T overexpression stable cell line using 1 μM of JTE 013 [S1PR2/HEK293T (-1)], 10 μM of JTE 013 [S1PR2/HEK293T (-10)], 1 μM of CYM-5520 [S1PR2/HEK293T (1)], 10 μM of CYM-5520 [S1PR2/HEK293T (10)] and control (DMSO treatment); **(E)** cAMP concentration determination by ELISA methods in the treatment of various concentration of berberine (0, 10, 20, 30, 40, 50 60, 70,80, 90, 100, 110, 120, 130, 140 μM); **(F)** mRNA expression level of RhoA, ROCK, ZO-1, E-cadherin and Occludin in the colon tissue samples; **(G)** Representative images of colon tissues in the BerM, BerL and BerH group by transmission electron microscopy.

In addition to regulating the gut bacterial community and bile acid composition to maintain a healthy balance, berberine also had the potential to promote the intestinal barrier repair. Based on the transcriptomics result, the expression level of S1PR2 was higher in the BerM group than that in the berberine treatment group. It was reported that the function of S1PR2 was related to maintaining the intestinal barrier (Chen et al., 2018; Chen et al., 2022). Thus, we constructed the S1PR2/HEK293T overexpression stable cell line to evaluate the inhibitory effect on the cAMP production activity of S1PR2 (Figure 7A). We initially detected the gene and protein expression level of S1PR2 in the stable cell lines through qPCR and Western blot method, and results showed that the expression level of S1PR2 in the S1PR2/HEK293T overexpression stable cell line was higher than that in the HEK293T and NC/HEK293T cell lines (Figures 7B, C; Supplementary Figure S1). We further used different concentrations of JTE 013 (antagonist for S1PR2) and CYM-5520 (agonist for S1PR2) to stimulate S1PR2/HEK293T overexpression stable cell line and observed that the cAMP production was inhibited and promoted by the JTE 013 (1 μM) and CYM-5520 (10 μM), respectively (Figure 7D). Then, a series concentration of berberine solutions were used to investigate. Results showed that 90 μM berberine showed the strongest inhibitory effect on the cAMP production activity of S1PR2/HEK293T stable cell line (Figure 7E). We also indicated that berberine inhibited the downstream RhoaA/ROCK pathway of S1PR2. Moreover, berberine might alleviate UC in the DSS induced model through increasing the mRNA expression of E-cadherin, Occludin, and ZO-1, which were the main tight junction proteins in colon tissue (Figure 7F). The transmission electron microscopy results also supported this opinion (Figure 7G).

## 4 Discussion

In recent years, an increasing number of studies have reported the role of bile acid metabolism in the development of various diseases. Du et al. (2024) explored the pathogenetic mechanism of butylparaben and observed that oral of this compound induced glucose intolerance and hyperlipidemia in mice model. They indicated that butylparaben inhibited the expression of FXR and decreased the relative abundances of Bacteroidetes, *Lactobacillus*, and *Streptococcus*, meanwhile, it affected the bile acid metabolism. Feng et al. (2024) analyzed the bile acid differences in individuals with type 2 diabetes mellitus coupling with non-alcoholic fatty liver disease. Results showed that the bile acid level in the disease group was lower than that in the healthy control group. In the United States, Ali et al. (2024) determined the bile acid level in the serum of patients with hepatitis C-related liver disease and observed that taurine-conjugated bile acids increased in the disease group. Moreover, the expression of S1PR2 was closely related to bile acids. Based on the above studies, the interaction between bile acid and target has been considered crucial for certain diseases, thus, the intervention of the dysfunction of bile acid metabolism using drugs offers a new idea for treating disease. Huang et al. (2024) investigated the treatment mechanism of dicaffeoylquinic acid for diabetic. They demonstrated that this natural product promoted the enterohepatic circulation of conjugated bile acids and inhibited the FXR-fibroblast growth factor 15 (FGF15) signaling axis in the ileum. Furthermore, it decreased the AMP-activated protein kinase pathway to ameliorate hyperglycemia and suppress inflammation. Liu et al. (2024) explained the mechanism of modified Gegen Qinlian decoction for type 2 diabetes mellitus and proposed the intestinal flora-bile acid-TGR5 axis might be the main intervention pathway. In this study, we used multi-omics technology to study the mechanism of the treatment effect of berberine on the UC in the DSS-induced model through the gut microbiome-bile acid-S1PR2 axis. Based on our results, *Duncaniella*, *Bacteroides*, and *Muribaculum* were dominant at the genus level. The relative abundances of *Duncaniella dubosii*, *B. caecimuris*, and *Muribaculum gordoncarteri* were higher than other species. Among these bacteria, certain of them were reported as probiotics in the previous reports (Chen et al., 2024; Yin et al., 2024; Zhang et al., 2024). Thus, berberine increased the relative abundances of beneficial bacterial community and inhibited the colonization of pathogens. Berberine also affected the metabolism of bile acid. We observed that there were significant differences of the bile acid level between the liver and colon tissue. Most bile acids showed an increasing trend from the liver tissue to the colon tissue, which gut microbiome might make great contributions during this process, for example, the bile salt hydrolase associated bacterial community (Firrman et al., 2024; Han et al., 2024; Rowe and Winston, 2024). Additionally, the taurine conjugated bile acids level was higher in the model group than that in the berberine treatment and control groups, which was similar to the conclusion of Schirmer et al. (2024). Common taurine conjugated bile acids include taurocholic acid (TCA), taurochenodeoxycholic acid, taurolithocholic acid, and taurodeoxycholic acid. Previous studies have reported that certain taurine conjugated bile acids might reflect the severity of disease and influence its development. The changes of bile acid composition in serum might cause cardiovascular disturbances in cirrhosis. Wiese et al. (2023) collected 15 individual serum samples to investigate the bile acid composition and indicated that TCA level correlated closely with hepatic venous pressure gradient (*p* = 0.01). Hertel et al. (2019) demonstrated that taurine-conjugated bile acids reflected the severity of motor symptoms, and low levels of sulfated taurolithocholate were related to Parkinson’s disease incidence. Zheng et al. (2022) analyzed the role of TCA and the Na^+^-taurocholate cotransporting polypeptide in orthotopic implantation model fed either regular or high-fat diet. Results showed that TCA promoted liver metastases and caused a tumor-favorable microenvironment in the liver, increasing proportions of myeloid-derived immune cells. Additionally, Lee et al. (2024) indicated that tauroursodeoxycholic acid had the potential to protecting against radiation-induced intestinal damage and inhibiting tumor cell migration without any radiation and radiation therapy effect through decreasing the inflammatory cytokine levels and endoplasmic reticulum stress in the irradiated intestinal cells. Bile acids affect disease development through directly regulating the host targets. We mainly focused on the activity of S1PR2, which was a crucial bile acid response GPCR in UC, and its downstream RhoA/ROCK pathway (Cao et al., 2024; Islam et al., 2024; Yang et al., 2023). Transcriptomics sequencing data demonstrated that the expression level of S1PR2 and RhoA/ROCK was higher in the model group than that in berberine treatment group. Moreover, experiment results showed that berberine inhibited the cAMP production of S1PR2 and the activation of RhoA/ROCK pathway, promoting the repairment of intestinal barrier dysfunction. However, it still requires further studies to deepen our understanding the interaction between gut microbiome, bile acids, and S1PR2 during the development of UC. On one hand, to our knowledge, the protein crystal structure of S1PR2 has not yet been resolved, which impedes the function analysis and drug development of this GPCR. On the other, the single target study was insufficient to explain the pathogenetic mechanism of UC. It has been reported that all five numbers in the S1PR family (S1PR1- S1PR5) make contributions in UC (Argollo et al., 2020; Wang et al., 2022). Thus, discussing the relationship and role of five S1PRs during the UC incidence and relapse may provide references for the new drug development of UC.

## 5 Conclusion

In this study, the protective effect of berberine on UC was investigated *in vitro* and *in vivo* models through multi-omics technologies (metagenomics, metabolomics, and transcriptomics). Based on our results, berberine had the potential to regulating the gut microbiome and metabolism of bile acid to alleviate the development of UC. On one hand, it inhibited the colonization of harmful bacteria and increased the relative abundances of beneficial bacterial such as *Akkermansia* and *Bacteroides*. Moreover, berberine inhibited the accumulation of taurine conjugated bile acid to suppress UC. On the other, berberine restored the intestinal barrier function through bile acid/S1PR2/RhoA/ROCK pathway. This study applied multi-omics technologies to assess the effect of berberine on UC, which provides a comprehensive insight to explore the mechanism of natural products on UC.

## Data Availability

The animal transcriptome and metagenome data were deposited in the NCBI repository, accession numbers PRJNA1139287 and PRJNA1139349 - available at https://www.ncbi.nlm.nih.gov/search/all/?term=PRJNA1139287 and https://www.ncbi.nlm.nih.gov/search/all/?term=PRJNA1139349. The metabolome data were deposited in the OMIX, China National Center for Bioinformation/Beijing Institute of Genomics, Chinese Academy of Sciences, accession numbers OMIX006898 and OMIX006897 - available at https://ngdc.cncb.ac.cn/omix/release/OMIX006898 and https://ngdc.cncb.ac.cn/omix/release/OMIX006897.

## Supplementary material

SUPPLEMENTARY FIGURE S1 Protein expression level of S1PR2 in the S1PR2/HEK293T overexpression stable cell line, GFP/HEK293T stable cell line, and HEK293T cell line in original WB results.
