# Supplementary figures and images for: Multi-omics reveals the alleviating effect of berberine on ulcerative colitis through modulating the gut microbiome and bile acid metabolism in the gut-liver axis
Source: Front Pharmacol. 2024 Oct 24;15:1494210. doi: 10.3389/fphar.2024.1494210 (PMC11540792; doi:10.3389/fphar.2024.1494210)

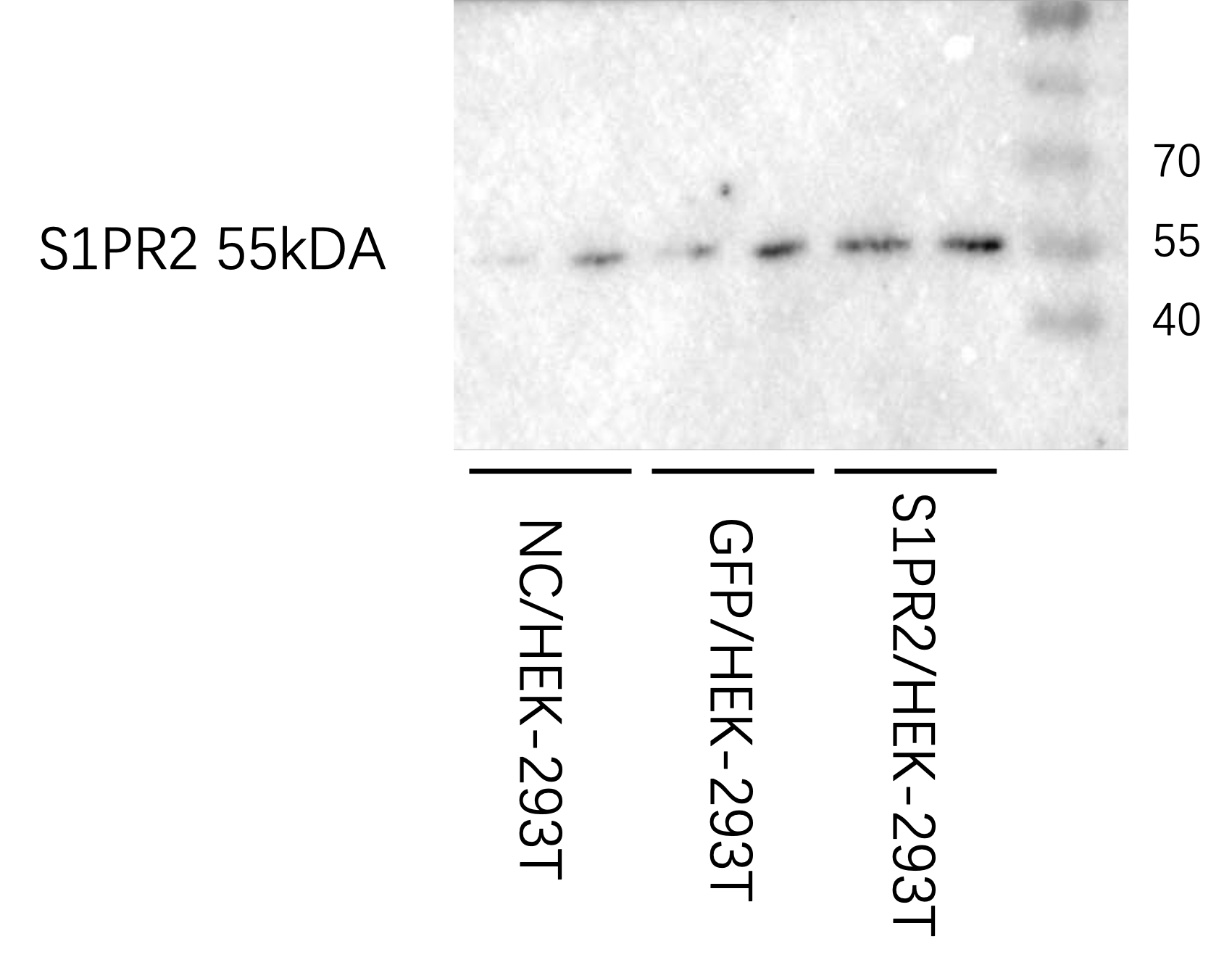

Supplement: Supplementary file 1 [file Image1.TIFF]
